# Analysis of the Putative Role of CR1 in Alzheimer’s Disease: Genetic Association, Expression and Function

**DOI:** 10.1371/journal.pone.0149792

**Published:** 2016-02-25

**Authors:** Maria I. Fonseca, Shuhui Chu, Aimee L. Pierce, William D. Brubaker, Richard E. Hauhart, Diego Mastroeni, Elizabeth V. Clarke, Joseph Rogers, John P. Atkinson, Andrea J. Tenner

**Affiliations:** 1 Department of Molecular Biology and Biochemistry, University of California Irvine, Irvine, California, 92697, United States of America; 2 Department of Neurology, University of California Irvine, Irvine, California, 92697, United States of America; 3 UCI Institute for Memory Impairment and Neurological Disorders, University of California Irvine, Irvine, California, 92697, United States of America; 4 SRI International, Menlo Park, California, 94025, United States of America; 5 Division of Rheumatology, Department of Medicine, Washington University School of Medicine, St. Louis, Missouri, 63110, United States of America; 6 Banner Sun Health Research Institute, Sun City, Arizona, 85351, United States of America; 7 School for Mental Health and Neuroscience (MHeNS), Department of Psychiatry and Neuropsychology, Faculty of Health, Medicine and Life Sciences, European Graduate School of Neuroscience (EURON), Maastricht University Medical Centre, Maastricht, The Netherlands; 8 Department of Neurobiology and Behavior and Department of Pathology and Laboratory Science, University of California Irvine, Irvine, California, 92697, United States of America; Colorado State University, College of Veterinary Medicine and Biomedical Sciences, UNITED STATES

## Abstract

Chronic activation of the complement system and induced inflammation are associated with neuropathology in Alzheimer’s disease (AD). Recent large genome wide association studies (GWAS) have identified single nucleotide polymorphisms (SNPs) in the C3b/C4b receptor (CR1 or CD35) that are associated with late onset AD. Here, anti-CR1 antibodies (Abs) directed against different epitopes of the receptor, were used to localize CR1 in brain, and relative binding affinities of the CR1 ligands, C1q and C3b, were assessed by ELISA. Most Abs tested stained red blood cells in blood vessels but showed no staining in brain parenchyma. However, two monoclonal anti-CR1 Abs labeled astrocytes in all of the cases tested, and this reactivity was preabsorbed by purified recombinant human CR1. Human brain-derived astrocyte cultures were also reactive with both mAbs. The amount of astrocyte staining varied among the samples, but no consistent difference was conferred by diagnosis or the GWAS-identified SNPs rs4844609 or rs6656401. Plasma levels of soluble CR1 did not correlate with diagnosis but a slight increase was observed with rs4844609 and rs6656401 SNP. There was also a modest but statistically significant increase in relative binding activity of C1q to CR1 with the rs4844609 SNP compared to CR1 without the SNP, and of C3b to CR1 in the CR1 genotypes containing the rs6656401 SNP (also associated with the larger isoform of CR1) regardless of clinical diagnosis. These results suggest that it is unlikely that astrocyte CR1 expression levels or C1q or C3b binding activity are the cause of the GWAS identified association of CR1 variants with AD. Further careful functional studies are needed to determine if the variant-dictated number of CR1 expressed on red blood cells contributes to the role of this receptor in the progression of AD, or if another mechanism is involved.

## Introduction

The complement system is a powerful effector mechanism of the innate immune system that contributes to protection from infection and autoimmunity as well as to resolution of injury [1]. However, if uncontrolled, inflammation resulting from ongoing and/or chronic complement activation can lead to tissue damage as seen in arthritis, age-related macular degeneration, traumatic brain injury and perhaps Alzheimer’s disease [1,2]. Recent GWAS have provided strong evidence for complement receptor CR1 being a risk factor for the development of AD [3–6]. Although, results from analysis of integrated functional network systems have consistently pointed to involvement of immune system pathways, particularly complement and inflammatory cytokines [7], the mechanistic basis for the CR1 risk remains unknown.

**Fig 1:**
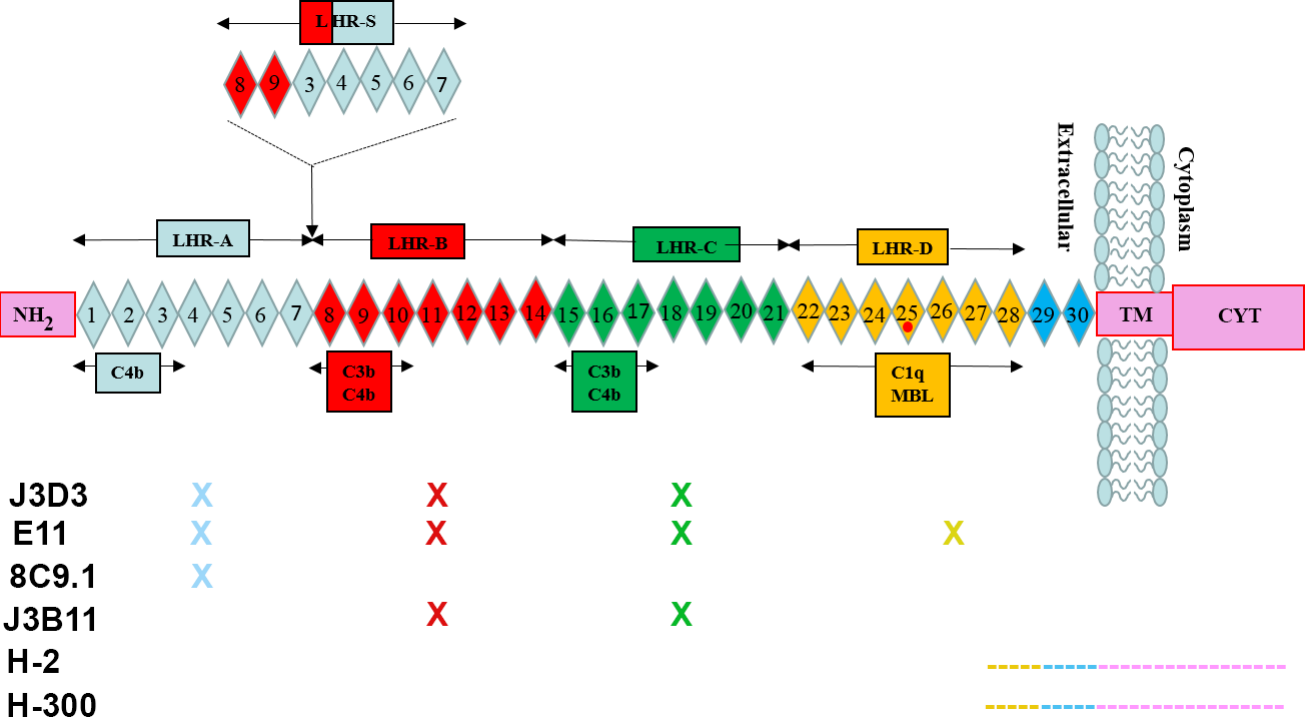
Representation of CR1 structure, ligand binding sites and location of antibody epitopes. CYT: cytoplasmic domain, TM: transmembrane domain. Red dot indicates location of rs4844609 (Ser1610Thr). Diagram modified from[16]; Antibody reactivity modified from[17].

CR1 is a transmembrane protein that enhances phagocytosis of particles opsonized with C3b, C4b, C1q, mannose-binding lectin (MBL) and ficolins, and, in primates, facilitates clearance of C3b-opsonized immune complexes via binding to red cell CR1 for trafficking to liver and spleen for disposal (known as the immune adherence reaction) [8–11]. In addition, CR1 suppresses the amplification of the complement cascade by both disrupting the C3 cleaving enzyme complex (C3 convertase) and by providing cofactor function to Factor I which cleaves C3b to a form that no longer can assemble a functional C3 convertase (reviewed in [12]). Three types of polymorphisms have been characterized in CR1: those that generate size variants, those that result in copy number differences on red blood cells (RBC), and those that generate the Knops blood group antigens [11,13]. The structure of human CR1 consists of 3–6 long homologous repeats (LHR), each containing seven short complement control protein motifs (CCP). The different size isoforms of CR1 result from the different number of LHRs as depicted in Fig 1 with the nomenclature and molecular weights provided in Table 1. The F (fast migration, CR1*1) and the S (slow migration, CR1*2) forms are the more common in the population (83 and 15% respectively) [11], with the largest CR1 (CR1*4) being rare (<1%) and the smallest (CR1*3or F’) having a gene frequency of 1 to 3%. The number of CR1 molecules on RBCs is correlated with a HindIII restriction fragment length polymorphism (RFLP). Homozygotes for low (L) copy number alleles can express fewer than 200 copies of CR1 per RBC while those homozygous for high (H) copy number alleles express 3–4 times more [11,13]. Further investigation showed that the HindIII polymorphism associated with the low expression allele was linked to three coding polymorphisms that resulted in Gln981His, His1167Arg and Pro1786Arg, suggesting that a lack of stability was the probable mechanism for the relationship of the HindIII restriction site and the expression of CR1 on RBC (reviewed in [13]). The importance of the Knops antigen polymorphisms remains to be determined [14].

**Table 1:** Electrophoretic mobility (Mr) and gene frequency of the allelic size isoforms of CR1.

Official name	Old name(s)	CR1 size (Mr) (non reduced)	CR1 size (Mr) (reduced)	N Number of LHRs	Frequency
CR1*4	**D**	**250,000**	**280,000**	**6**	**<0.01**
CR1*2	**B or S**	**220,000**	**250,000**	**5**	**0.15**
CR1*1	**A or F**	**190,000**	**220,000**	**4**	**0.83**
CR1*3	**C or F’**	**160,000**	**190,000**	**3**	**0.01**

Modified from [11]. Official names [18]. S, slow and F, fast, reflecting Mr on sizing gels.

Some of the functional binding domains have been assigned as shown in the diagram in Fig 1. rs6656401 is a SNP in a noncoding segment of the CR1 gene [3,15] and, as such, is associated with the inclusion of a fifth LHR domain (also referred to as the slow migrating (S) form (Table 1)), and thus it contains an additional C3b binding site in the protein.

The more recently discovered CR1 SNP associated with AD, rs4844609, is observed with a subset of the originally GWAS identified rs6656401 variants [3]. Thus far, it is the only CR1 SNP identified as associated with AD that is within the coding region of CR1 (CCP 25 of LHR-D) [16]. It coincides with a region of the molecule that has previously been implicated in binding to C1q and MBL [19] (Fig 1) and has recently been definitively shown as the region (CCP 24 and 25) where MBL and ficolins (which share structural properties with C1q) bind CR1 [10]. While a role for MBL in AD is currently unknown [20], C1q, C4b and C3b associate with fibrillar Aß [21–24].

Rogers and colleagues have suggested that Aß is cleared from blood via complement-dependent adherence to CR1 expressed on RBC [25]. Since it has been shown that C1q, C4b and C3b each bind to CR1 and additively support binding to erythrocyte CR1 [26], rs4844609 in association with re6656401 could have an impact on clearance of amyloid from the blood via C1q, C4b and/or C3b. It is also possible that the rs4844609 alteration (in CCP 25) may modulate access to the cleavage site that generates soluble CR1 (sCR1), which has been localized to CCP 29–30 [27]. Changes in the cleavage efficiency to generate sCR1, a molecule known to have complement inhibitory activity, could lead to less regulation of cell surface complement activation and may also affect immune complex/amyloid clearance [28,29]. There is, however, some controversy as to the identity of the AD-associated CR1 SNPs as the rs4844609 association was not found in another large cohort [30]. Those investigators postulated that the rs6656401 CR1 SNP represents the functional risk factor in AD.

Studies presented here demonstrate specific reactivity of two anti-CR1 mAb with astrocytes in the human brain but there is no association with diagnosis or the presence of the SNPs rs4844609 and 6656401. Assessment of sCR1 concentrations in fresh plasma showed only a slight increase in concentration in that from individuals with both SNPs (rs4844609 and 6656401). Binding interaction of C1q with the variant CR1s extracted from red blood cells showed small increases in relative binding of CR1 to C1q. In addition, C3b-CR1 binding increased in the genotypes containing the slow (S) form of CR1 (and thus the additional C3b binding site) with the rs6656401 SNP compared to the more common alleles. No correlation of these parameters, astrocyte expression or ligand binding, was linked to the clinical diagnosis.

## Materials and Methods

### Subjects

All human materials were acquired under approved Institutional Review Board (IRB) protocols for University of California, Irvine, Stanford Research Institute, Banner Sun Health Research Institute, and Northwestern University. These boards specifically approved studies of this nature in which all patient/donor identifiers are removed.Written informed consent from the donor or the next of kin was obtained for the use of the tissues reported in research as part of the tissue repositories. The human brain tissues (Table 2), plasma and red blood cells (Table 3), and DNA SNP analyses used in this project were provided by the University of California Irvine Alzheimer's Disease Research Center (UCI-ADRC), SRI International (SRI) and Banner Sun Health Research Institute (BSHRI). Human brain tissue (middle frontal gyrus and hippocampus) was collected within 1 to 13 h of death and was fixed in 4% paraformaldehyde or 10% formalin (used for immunohistochemistry (IHC)) or frozen (used for Western blots) by the Tissue Repository of the Institute for Memory Impairments and Neurological Disorders at the University of California, Irvine. Brain tissue used for astrocyte cultures was obtained through BSHRI (postmortem interval of 2.4 h). Subjects included in this study received ante-mortem evaluation by board-certified neurologists and postmortem evaluation by a board-certified neuropathologist. Evaluations and diagnostic criteria followed consensus guidelines for National Institute on Aging Alzheimer’s Disease Centers.

**Table 2:** Case Characteristics for Analyzed Brain Samples.

Case #	Sample	Age	Sex	Dx	APOE	rs6656401	rs4844609
**BN1**	**Brain**	**65**	**M**	**Normal**	**3/4**	**GG**	**TT**
**BN2**	**Brain**	**81**	**M**	**Normal**	**3/3**	**GG**	**TT**
**BN3**	**Brain**	**86**	**M**	**Normal**	**3/3**	**GG**	**TT**
**BN4**	**Brain**	**>89**	**F**	**Normal**	**2.3**	**GG**	**TT**
**BN5**	**Brain**	**86**	**F**	**Normal**	**3/3**	**AG**	**TT**
**BN6**	**Brain**	**89**	**F**	**Normal**	**3/3**	**AG**	**TT**
**BN7**	**Brain**	**>89**	**M**	**Normal**	**4/4**	**AG**	**TT**
**BN8**	**Brain**	**>89**	**M**	**Normal**	**3/3**	**AG**	**AT**
**BN9**	**Brain**	**79**	**M**	**Normal**	**2/4**		
**BD1**	**Brain**	**72**	**M**	**AD**	**4/4**	**GG**	**TT**
**BD2**	**Brain**	**73**	**M**	**AD**	**3/4**	**GG**	**TT**
**BD3**	**Brain**	**77**	**F**	**AD**	**3/4**	**GG**	**TT**
**BD4**	**Brain**	**81**	**F**	**AD**	**3/3**	**GG**	**TT**
**BD5**	**Brain**	**82**	**F**	**AD**	**3/4**	**GG**	**TT**
**BD6**	**Brain**	**>89**	**M**	**AD**	**3/3**	**AG**	**TT**
**BD7**	**Brain**	**>89**	**F**	**AD**	**3/3**	**AG**	**TT**
**BD8**	**Brain**	**77**	**F**	**AD**	**4/4**	**AG**	**AT**
**BD9**	**Brain**	**89**	**M**	**AD**		**AG**	**AT**
**BD10**	**Brain**	**76**	**F**	**AD**	**3/4**	**AG**	**AT**
**BD11**	**Brain**	**71**	**M**	**AD**	**3/3**	**AA**	**AT**
**BD12**	**Brain**	**79**	**M**	**AD**	**3/4**	**AA**	**TT**
**BD13**	**Brain**	**63**	**M**	**AD**	**3/4**		**AT**
**BD14**	**Brain**	**75**	**F**	**AD**	**3/3**		

**Table 3:** Characteristics for Analyzed Plasma and Reb Blood Cell CR1 Samples.

Sample type		# and gender	Age mean	Age range
**Plasma**	**UCI cohort**	**21 M**	**73**	**50–92**
**Plasma**	**UCI cohort**	**38 F**	**76**	**48–88**
**Red Blood Cells**	**UCI cohort**	**14 M**	**79**	**70–92**
**Red Blood Cells**	**UCI cohort**	**15 F**	**76**	**49–87**
**Red Blood Cells**	**SRI cohort**	**33 M**	**80**	**60–91**
**Red Blood Cells**	**SRI cohort**	**30 F**	**82**	**61–92**

### Tissue dissociation and primary cell culture

Postmortem brain astrocyte cultures were established following previously developed methods with minor modifications [31]. In brief, AD brain tissue was quickly transported in ice-cold Hibernate A medium (BrainBits, LLC; Springfield, IL), mechanically dissociated into 1–2 mm pieces, and digested with 0.25% trypsin (Irvine Scientific, Santa Ana, CA) and 0.1% DNase (Sigma, St. Louis, MO) in a shaking water bath at 30°C for 15 min. Digestion was stopped with fetal bovine serum (FBS). After passing the cell and tissue suspension through progressively finer metal screens, it was diluted with complete DMEM (high glucose, minus phenol red) (Invitrogen-Gibco, Carlsbad, CA) [containing 10% FBS (Gemini Bio-Products; West Sacramento, CA); 0.02M HEPES (Irvine Scientific); 1 mM sodium pyruvate (Mediatech Cellgro, Herndon, VA); penicillin/streptomycin (Invitrogen-Gibco); and gentamycin (Irvine Scientific)]. Cells and debris were separated using 50% Percoll-gradient (Amersham/GE Healthcare, Piscataway, NJ) and centrifugation (Sorvall RC6, SLA-1500 rotor, RCF 20,020 xg; 13,000 rpm; 4°C). The first layer of myelin and debris was discarded. The second layer of the gradient, which is rich in microglia and astrocytes was removed, washed, pelleted, gently triturated, washed a second time, resuspended in complete DMEM (plus phenol red), and transferred to a 75-ml tissue culture flask (Nunc, Rochester, NY). After 2–24 h at 37°C/7% CO_2_, nearly 98% of microglia became adherent [31], and thus culture supernatants that were relatively free of microglia could be transferred to a second set of 75-ml flasks for the plating of astrocytes. The secondary flasks were left undisturbed, except for weekly medium replacement with complete DMEM, for 1–3 weeks in tissue culture incubators maintained at 37°C with 7% CO_2_, after which cells in the supernatant were seeded as needed.

### Immunohistochemistry and Immunocytochemistry

Tissue sections (50 μm) (from cortex or hippocampus) were pretreated with 3% H_2_O_2_/10% MeOH/Tris Buffer Saline (TBS), pH 7.4 to block endoperoxidase. After blocking with 2% BSA/ 0.1% Triton/TBS, sections were incubated with different anti-CR1 Abs: mouse monoclonal antibodies 8C9.1 (1 μg/ml), J3B11 (3 μg/ml) [17], E11 (5 μg/ml, Abcam, Cambridge, MA, Cat # ab25,AB_448530), J3D3 (5 μg/ml, Beckman-Coulter, Cat # IM0195), clone #594708 (5 μg/ml, R&D, Cat #MAB5748,AB_10717677), H-2 (4 μg/ml, Santa Cruz, Cat# sc-166329, AB_2292232), rabbit polyclonal Ab against carboxyl-terminus (H-300, 4 μg/ml Santa Cruz,sc-20924,AB_2085023) or rabbit polyclonal Ab anti-CR1 1 μg/ml [32] in blocking solution, overnight at 4°C. Sections were next incubated with biotinylated horse anti-mouse Ab or goat anti-rabbit (Vector, Burlingame, CA) (1 h, RT (room temperature)) followed by ABC (Vector) (1 h, RT) and developed with DAB (Vector). Tissue was dehydrated and mounted with DePeX (BHD Laboratory Supplies, England). Specificity of staining was demonstrated by a lack of reactivity when using mouse IgG corresponding isotype (IgG1 or IgG2b) or rabbit IgG (at the same concentrations as the primary Ab) instead of primary Ab as controls. In addition, anti-CR1 mAbs 8C9.1 and J3B11 were preabsorbed with recombinant human CR1 (R&D, Minneapolis, MN) (1:15 molar ratio) before incubation with the tissue to validate specificity of staining.

For immunofluorescent colocalization of the anti-CR1 and GFAP (astrocyte marker), sections were incubated with anti-CR1 8C9.1 (1 μg/ml) and rabbit polyclonal anti bovine GFAP (Dako, 1:3000, Cat#Z0334,AB_2314535) overnight at 4°C, followed by Alexa 488 labeled anti-mouse and Alexa 555 labeled anti-rabbit secondary Abs (Invitrogen, Carlsbad, CA). Tissue was mounted with Vectashield (Vector). For colocalization of CR1 with Iba1 staining, both 8C9.1 antibody and Iba1 antibody (Wako, Cat# 019–19741, AB_839504) were used at 1 ug/ml. Secondary antibodies used were Alexa488 (green) anti mouse and Alexa555 (red) anti rabbit respectively.

CHO cells (ATCC CCL-61) expressing the CR1 receptor at 1 x 10^6^ or 1 x 10^5^ receptor/cell were plated on coverslips at 1 x 10^5^ cells/ml in 10% FCS/Hank’s F12 containing glutamine and the antibiotic G418 (200 μg/ml) for 24 h. Cells were fixed in 4% formaldehyde in PBS (phosphate buffered saline). Cells were blocked in 2% BSA/5% NGS (normal goat serum) /PBS for 1 h at room temperature (RT) and incubated with 2 μg/ml of anti-CR1 Abs: E11, J3B11, 8C9.1, #594708, H-2, pAb CR1 or the corresponding mouse or rabbit control IgGs for 1 h at RT. After washes, cells were incubated with Alexa 555 labeled goat anti-mouse Abs. Coverslips were mounted in Vectashield (Vector).

Pictures were taken under bright field or fluorescence optics with Zeiss Axiovert 200 (Carl Zeiss, Thornwood, NY). Images were captured with Zeiss Axiovision 4.6 software.

For immunocytochemical analysis of astrocyte cultures, cells were briefly washed with PBS and then fixed with acetone-ethanol (1:1) for 5 min at RT. After washing, nonspecific binding was blocked with either 3% NGS (Sigma)/PBS or 1% BSA (Sigma)/PBS (depending on the source of secondary Ab) for 45 min at RT. Cultures were then incubated with primary Abs 8C9.1 (6 μg/ml) and J3B11(3 μg/ml) diluted in PBS for 1 h at RT. Following three brief washes, cells were incubated with anti-mouse secondary Abs conjugated with Alexa Fluor 488 or Alexa-Fluor 594 fluorophores (Invitrogen/Molecular Probes) in the dark for 1 h at RT or in some cases overnight at 4°C. Colocalization experiments with anti GFAP were carried out by incubating cultures sequentially in two primary Ab solutions, each from a different species, followed by incubation with species-appropriate secondary Abs conjugated to Alexa Fluor 488 for one marker and Alexa Fluor 568 for the other marker. Immunostained cell cultures were examined on Olympus IX70 microscopes equipped with epifluorescence illumination and confocal laser scanning using argon and krypton lasers (IX70), and images captured with a Nikon DS-LI and Olympus DP-71 color digital cameras or, for confocal microscopy, by Fluoview software (Olympus). Contrast and brightness adjustments and overlay compositing were done with Adobe Photoshop CS3. No staining was observed when primary Abs were deleted.

### Western Blot (WB)

Brain tissue (cortex) was homogenized in TPER (Pierce) (0.150g tissue/ml) containing a cocktail of protease inhibitors (complete Mini) (Roche Diagnostics, Indianapolis, IA). Homogenates were centrifuged at 4°C, 15,000 g for 30 min. RBC membranes were isolated as previously described [25] with some modifications. Briefly, red blood cells (RBC), freshly isolated or shipped overnight, were centrifuged and lysed with 5 volumes of ice cold water with protease inhibitor cocktail (PIC, Roche Diagnostics, Indianapolis, IN) at 4°C for 30 min. Pelleted membranes were extracted in 1% NP40 in 50 mM Tris/150 mM NaCl/1 mM EDTA with protease inhibitors and centrifuged at 15,000 g for 30 min. Protein concentration in the supernatants was determined with BCA protein assay (Pierce, Rockford, IL). CR1 concentration in RBC extracts was determined by ELISA (see below). Alternatively (SRI), CR1 was partially purified from membrane samples by ion-exchange chromatography, with all purification steps being carried out at 4°C and pre-chilled buffers in the presence of 10 mM acetate pH 5.0 and 1x PIC. Membranes were solubilized in 1% ASB-14 (Sigma-Aldrich) and 100 mM NaCl, rotated at 4°C for 30 min, followed by a 5 min spin at 17,000 x g. The supernatant was diluted 1:10 in 50 mM NaCl, 0.1% ASB-14, and applied to a HiTrap SP HP 1 ml (GE Healthcare 17-1151-01) cation-exchange column equilibrated at 50 mM NaCl. After washing with 5 column volumes of buffer at 50 mM NaCl + 0.1% ASB-14 and 5 column volumes of 250 mM NaCl + 0.1% ASB-14 to remove hemoglobin and other contaminants, sample was eluted in 1.0 M NaCl, 0.1% ASB-14. The buffer was exchanged to 10 mM Tris, 100 mM NaCl, 0.1% ASB-14, and 1x PIC and concentrated using 15 mL centrifugal ultrafiltration units (Pierce) with a Mol wt cut -off of 150 kDa. CR1 was dispersed into aliquots, flash-frozen on dry ice, and stored at -70°C.

Brain extracts (100 μg protein per lane) or RBC extracts (CR1 ~ 0.9 ng) were run on 7.5% SDS (sodium dodecyl sulfate)-polyacrylamide gel under nonreducing conditions. Proteins were transferred to polyvinylidenedifluoride (PVDF, Millipore Corporation, Bedford, MA) membrane (350 mA for 3 h). Membranes were blocked with 3% nonfat dry milk in 0.1%Tween/TBS for 1 h and then incubated overnight at 4°C with primary Abs (E11, 8C9.1, J3B11, H-2 or polyclonal anti-CR1 [32]). After washing, blots were incubated with HRP-labeled anti-mouse or anti-rabbit secondary Abs (1:10,000) (Jackson Immunoresearch, West Grove, PA) for 1 h. Labeling was detected using the ECL system (Amersham Biosciences or GE Healthcare). For cross reactivity testing of CR1 antibodies with CR2 by WB, recombinant CR2 and anti CR2 monoclonal Ab (MAB4909)were from R&D.

### Immunoprecipitation (IP)

RBC extracts (50 μg) or brain extracts (250 μg), prepared as indicated above, or soluble recombinant human CR1 were incubated with 4 μg of anti-CR1 Abs E11, 8C9.1 or J3B11 (in 1%NP40/TBS/EDTA buffer containing protease inhibitors) overnight at 4°C in rotator. Next, 20 μl of Protein G Dynabeads (Life Technology) were added and samples were incubated for 2 h at 4°C in rotator. Beads were separated with a magnet and washed three times with incubation buffer. Beads were resuspended in elution buffer (50 mM glycine, pH 2.8) for 1 min and 4X nonreduced SDS sample buffer was added. Samples were boiled for 5 min. After separation of beads with a magnet, supernatant was loaded in a 7.5% polyacrylamide gel and WB was performed as indicated above.

### Determination of concentration of sCR1 in plasma and CR1 in extracts from RBC

Immulon2HB plates (Thermo Fisher-Hyclone) were coated with J3D3 CR1 mAb at 0.2 μg/ml in PBS, overnight at 4°C. Remaining sites were blocked with PBS containing 3% BSA (Sigma) for 1 h at RT. rhCR1, plasma or CR1 isolated from RBC as described above for WB were diluted in PBS/1% BSA, 0.1% n-Octyl-beta-D-glucopyranoside (OG) (Calbiochem), added to Ab coated wells in triplicate, and incubated on a rotator for 1 h at RT. Wells were washed with PBS/0.01% OG prior to addition of E11 biotinylated anti-CR1/CD35 at 0.8 μg/ml (Ancell) in PBS/1% BSA, 0.1% OG and incubated on a rotator for 1 h at RT. After washing, streptavidin-HRP (Invitrogen) was added at 0.25 μg/ml for 30 min on a rotator at RT, followed by Ultra TMB (Thermo Scientific) and then 2 M H_2_SO_4_ to stop development of TMB. CR1 or sCR1 was assessed by measurement of the absorbance at 450 nm, and the average value of triplicates compared to a standard curve generated with known concentrations of rhCR1.

### ELISA for determining the relative binding activity of CR1 to C3b and C1q

Immulon2HB plates were coated with C3b or BSA at 2.5 μg/ml or C1q or BSA at 10 μg/ml in PBS overnight at 4°C. Remaining sites were blocked with PBS containing 3% BSA for 1 h at RT. Standard protein rhCR1, CR1 isolated from RBC, secondary Ab biotin-E11 and streptavidin-HRP were diluted in 0.01 M Tris pH 7.5 /0.1 M NaCl /1% BSA, 0.1% OG, 0.01% Amidosulfobetaine-14 (ASB-14) for C3b-CR1 ELISA. Dilutions for the C1q-C3b binding assay were in the same buffer except for the omission of ASB-14 and the optimized pH of 7.2. After addition of the CR1 samples, incubation was for 1 h at RT. Wash buffer was 0.01 M Tris /0.1 M NaCl /0.01% OG (pH 7.5 for C3b-CR1 ELISA or pH 7.2 for C1q-CR1 ELISA). All remaining steps and reagents to develop the assay were the same as described above. Binding of CR1 was assessed by measurement of the absorbance of triplicate wells at 450 nm. Relative binding activity values were analyzed from the slope (OD_450_ vs sample concentration in the linear range from 0–2.8 pM for C3b or 0–1.8 pM for C1q) and then standardized by reference samples in each assay. Diagnosis of the sample donors was based on clinical assessment at the time of blood collection.

## Results

### CR1 colocalizes with astrocytes in brain

**Fig 2:**
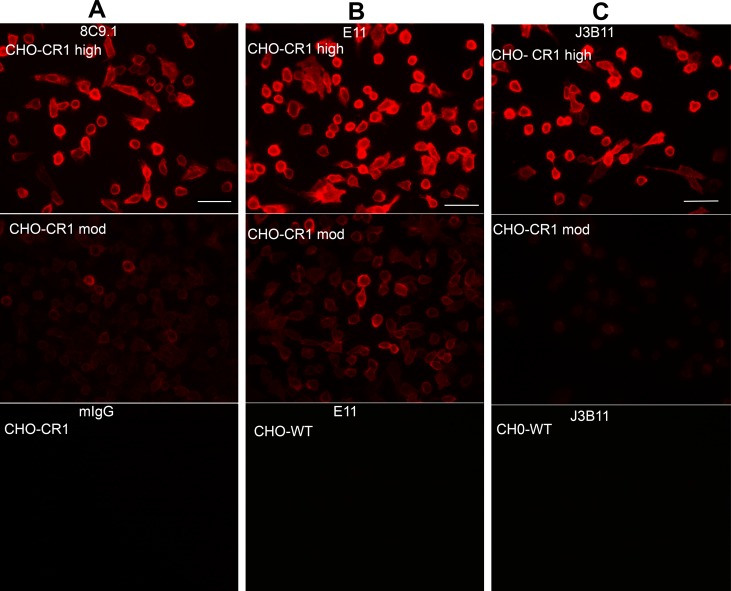
CR1 expressed by CHO cells is recognized by anti-CR1 antibodies. CR1 immunofluorescence staining using mouse mAbs 8C9.1(A), E11 (B) or J3B11 (C) on fixed CHO cells expressing 1x10^6^ (high) (upper panels) or 1x10^5^ (moderate) (middle panels) receptors/cell. Bottom panels show isotype control on high CHO-CR1 (A), or antibody reactivity on non-transfected CHO cells (B, C). Scale bar: 50 μm.

To assess the expression of CR1 in AD brain by CR1 immunoreactivity, multiple Abs directed against different epitopes of CR1 (Fig 1) were acquired. Antibody specificity was initially verified by reactivity employing CHO cells expressing CR1. The monoclonal anti-CR1 Abs tested, 8C9.1, E11, J3B11 and #594708, and the one polyclonal reacted similarly, giving a fluorescence signal in CHO cells expressing 1x10^6^ receptors/cell (Fig 2A, 2B and 2C upper panels and data not shown) and dimmer staining in cells expressing 1x10^5^ receptors/cell (Fig 2A, 2B and 2C middle panels and data not shown). H-2 mAb however, did not stain CHO-CR1 cells (S1 Table). CHO cells that were not transfected with CR1 showed no staining indicating specificity of antibody reactivity (Fig 2B and 2C lower panels and data not shown). Mouse IgG of the same isotype of the anti-CR1 Abs was used as a negative control and did not give any staining (Fig 2A lower panel and data not shown). These antibodies do not crossreact with CR2 as tested by immunocytochemistry using K562 cells expressing CR2 with an anti human CR2 antibody as a positive control. In addition, purified CR2 was not recognized by J3B11 by WB while anti CR2 did react specifically with CR2 but not with CR1 in parallel lanes. (Data not shown).

**Fig 3:**
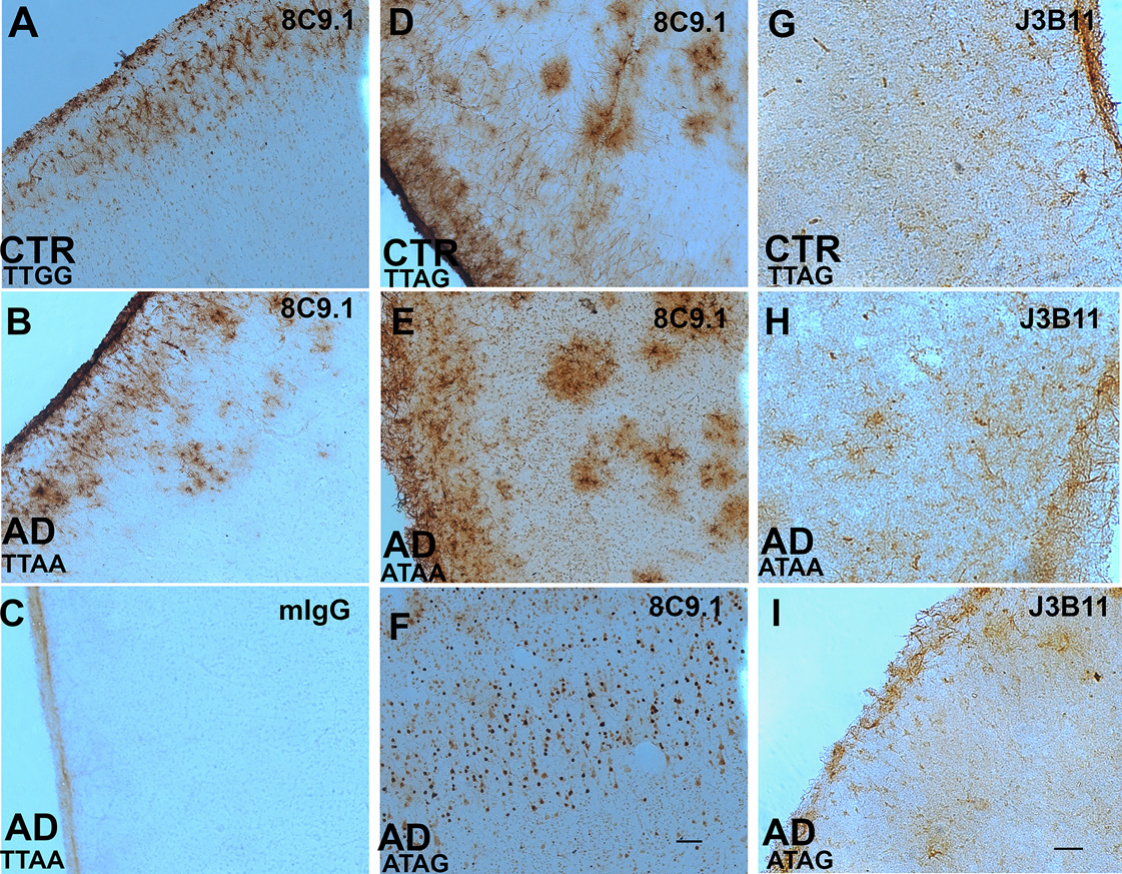
CR1/CD35 expression in human brain. Representative micrographs of brain sections stained with monoclonal anti-CR1 Abs 8C9.1 (A, B, D-F), J3B11 (G-I) or mIgG control (C) in frontal cortex of control (CTR) and Alzheimer’s disease (AD) cases. Genotype for the two GWAS SNPs rs4844609 (T or A) and rs6656401 (G or A) are presented in each panel. Scale bar 100 μm.

In brain sections, monoclonal anti-CR1 Abs 8C9.1 (Fig 3A, 3B and 3D–3F) and J3B11 (Fig 3G–3I) labeled cell bodies and processes of cells with the characteristic morphology of astrocytes in all 9 nondemented cases and 14 AD cases (Table 2). The cases studied included 6 with and 15 without the rs4844609 SNP (plus two unknown genotypes), 11 with and 9 without the rs6656401 SNP (three unknown genotypes) and 11 with and 8 without the rs3818361 SNP (Table 2).

**Fig 4:**
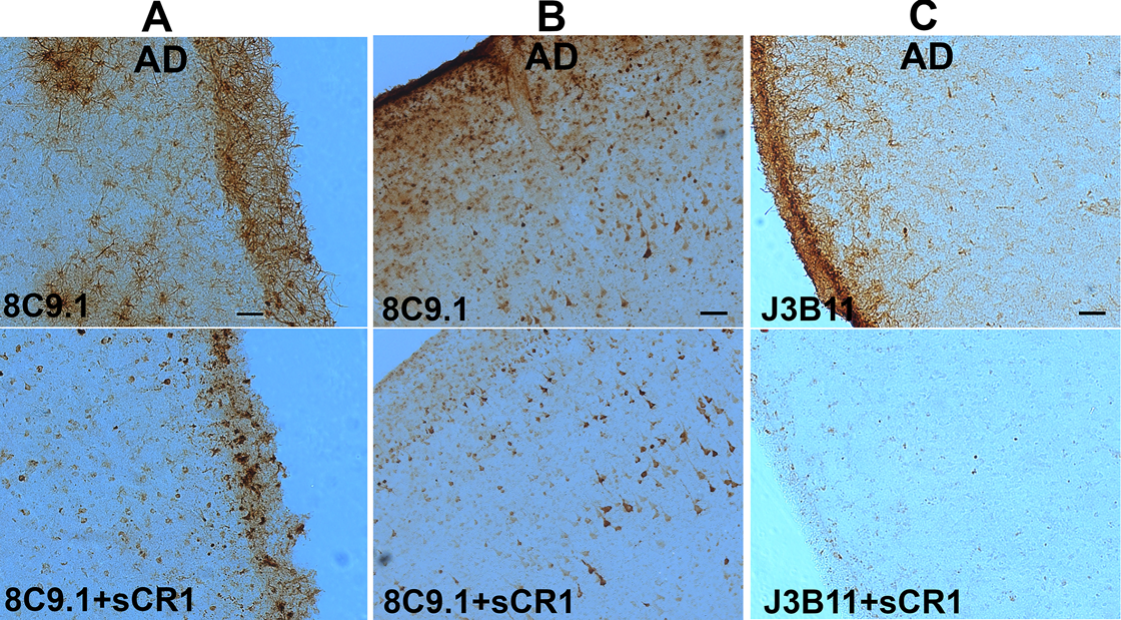
Anti-CR1 immunoreactivity with astrocytes but not neurons is preabsorbed by rhCR1. Immunostaining with monoclonal anti-CR1 8C9.1 (A, B) or J3B11 (C) without (upper panels) or with (lower panels) preabsorption with recombinant human CR1. Neuronal staining (B) was not preabsorbed with rhCR1. Scale bar: A, C: 50 μm, B: 100 μm.

In general, the staining was present in all cell layers of cortex and was abundant in the external layers of cortex including a thick network of astrocyte processes in the pial/glial limitans. The extent of astrocyte staining was variable in the different cases studied and not associated with a particular diagnosis or CR1 SNP. Pre-absorption of Abs with human recombinant sCR1 (but not BSA) blocked astrocyte staining by 8C9.1 (Fig 4A and 4B) and J3B11 (Fig 4C). Some neuronal nuclear staining was also seen in 7 of 23 cases, both nondemented and AD (Fig 3F, and Fig 4) with 8C9.1. However, this reactivity was not preabsorbed with recombinant sCR1 (Fig 4A and 4B), suggesting that the staining in neurons was due to Ab cross reactivity with another molecular structure. No staining was seen when isotype control IgG was used as the primary Ab (Fig 3C and data not shown).

**Fig 5:**
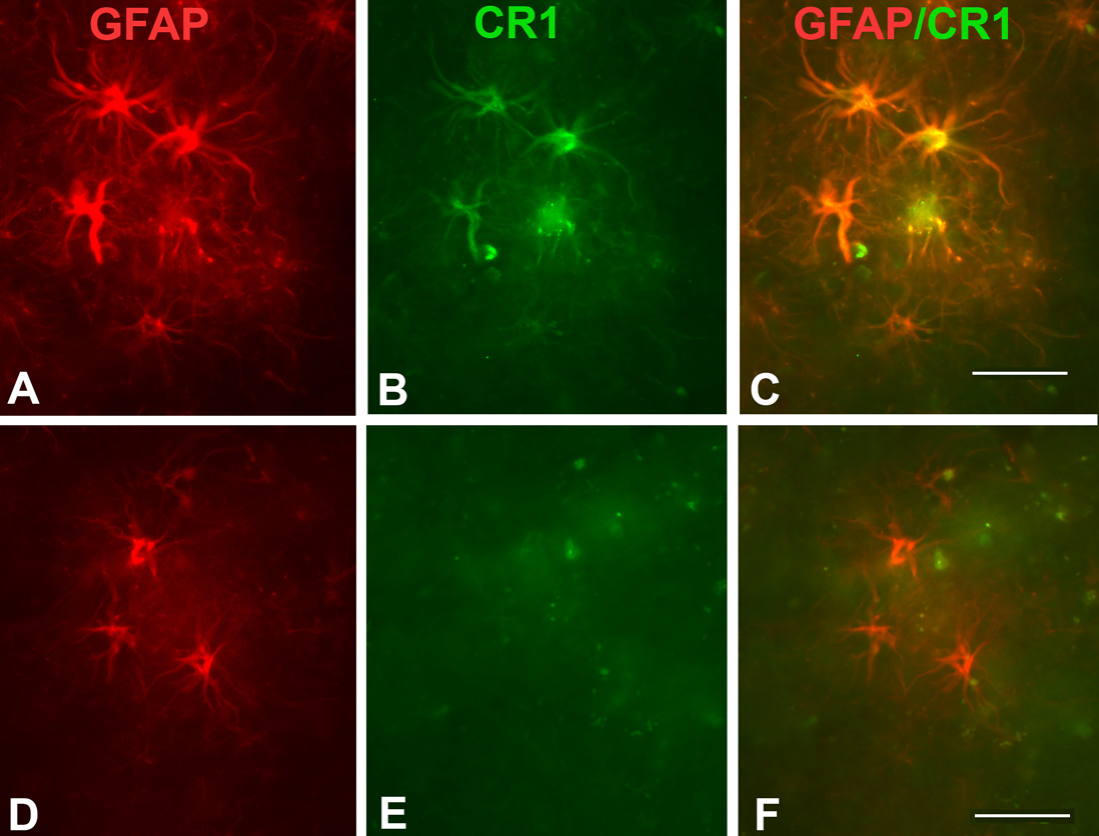
CR1 staining colocalizes with a subset of astrocytes. Immunofluorescent dual labelling of CR1 (8C9.1, red) and astrocytes (anti-GFAP, green) in an AD case (A-F). Merged images show colocalization (C) or lack of colocalization (F) of CR1 and GFAP immunofluorescence labelling. Scale bar: 50 μm.

Colocalization of clone 8C9.1 reactivity with GFAP confirmed that the CR1 staining was present in astrocytes (Fig 5A–5C), although not all astrocytes were positive for CR1 (Fig 5D–5F), particularly towards the deeper layers of the cortex. Immunofluorescent dual labelling using 8C9.1 and Iba1 antibodies showed that CR1 staining does not colocalize with microglia. (S1 Fig).

**Fig 6:**
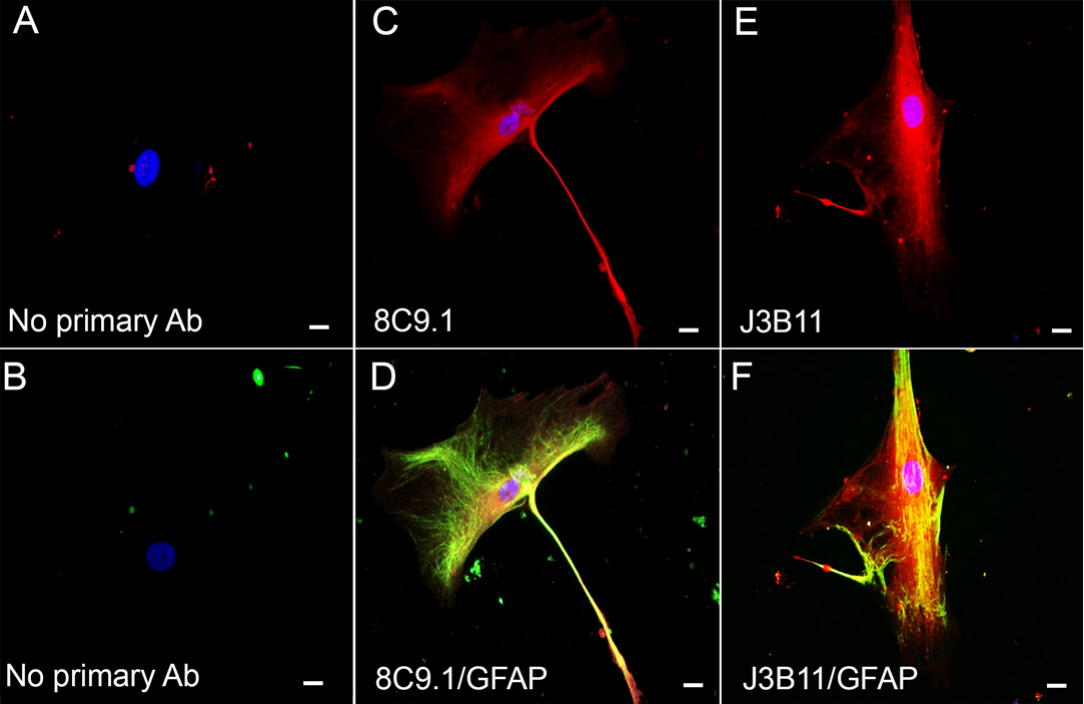
CR1 expression by human AD brain-derived astrocytes. Representative confocal photomicrographs of astrocytes isolated from post-mortem human brain stained with 8C9.1 (red) (C, D) and J3B11 (red) (E, F) mAbs and anti-GFAP (green) (D, F). A, B negative controls in which only secondary Ab labeled with Alexa555 (A) or Alexa488 (B) but no primary antibodies were added. Scale bar 20 μm.

Astrocyte staining with both mAbs was also supported by immunocytochemistry and colocalization with GFAP in human-derived astrocyte cultures (isolated from an AD Braak VI case). Cultured astrocytes contained relatively large somas (50–70 µm) and other common morphologic characteristics of type 1 astrocytes (Fig 6C and 6E) [33–35]. Both mAbs 8C9.1 (Fig 6C) and J3B11 (Fig 6E) showed diffuse immunoreactivity throughout the cell soma that colocalized with GFAP (Fig 6D and 6F). While membrane expression was expected, nonactivated neutrophils also show intracellular CR1 [36]. In contrast, microglia derived from adult post mortem human brain were stained with Iba-1 and anti CD11b, but not with 8C9.1 or J3B11 (data not shown).

Anti-CR1 mAb E11 and J3D3 and pAb against a carboxyl-terminal epitope (Santa Cruz) recognized CR1 on RBC in blood vessels of brain, but did not stain neurons or glial cells (data not shown). mAb from R&D (clone #594708) was completely negative in brain by IHC. One rabbit polyclonal and the H-2 monoclonal anti-CR1 gave neuronal staining that was not preabsorbed with sCR1 in multiple brain tissues tested (data not shown). Finally, no staining of the choroid plexus was observed with any of the antibodies tested (data summarized in S1 Table).

### Anti-CR1 mAb 8C9.1 and J3B11 detect CR1 in lysates from red blood cells but not brain

**Fig 7:**
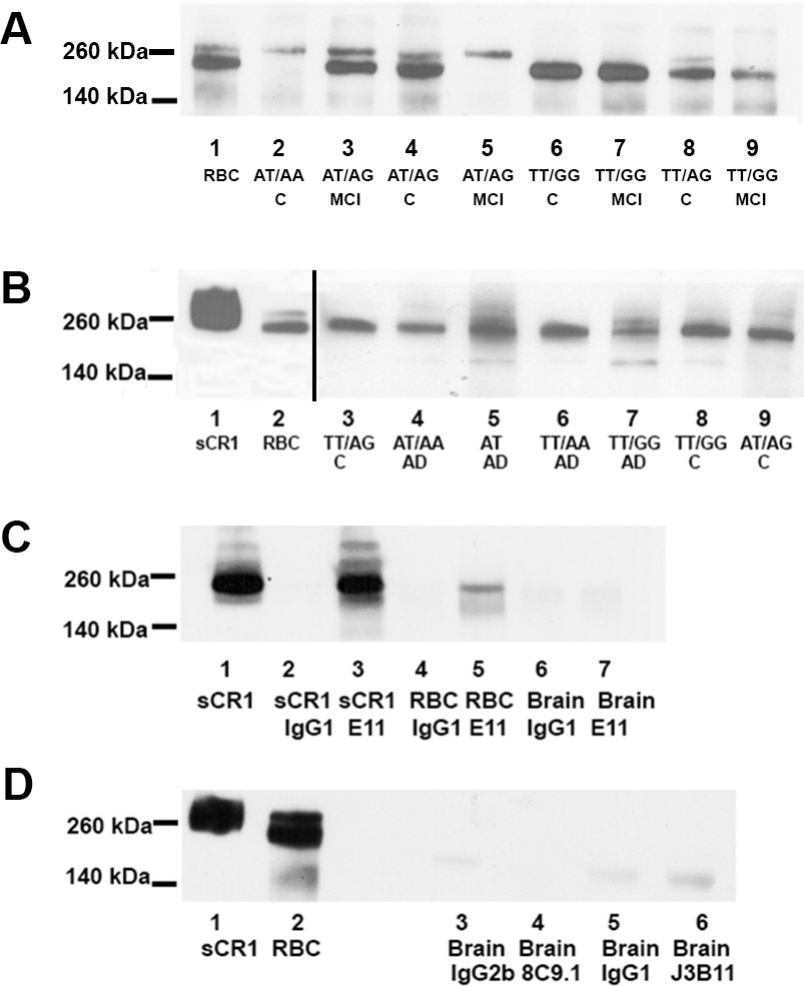
WB and immunoprecipitation of CR1 with mAbs E11, 8C9.1 and J3B11. (A) WB of RBC samples with and without SNP rs6656401 (loaded 0.9 ng CR1 per lane) using E11 mAb. Lanes: 1 RBC control, 2 C (nondemented) (AT/AA), 3 MCI (mild cognitive impairment) (AT/AG), 4 C (AT/AG), 5 MCI (AT/AG), 6 C (TT/GG), 7 MCI (TT/GG), 8 C (TT/AG), 9 MCI (TT/GG). (B) WB of brain extracts from donors with and without rs6656401 (loaded 100 μg brain extract protein per lane 3–9) probed with E11 mAb. Lane: 1 sCR1 (1 ng), 2 RBC (5 μg), 3 C (TT/AG), 4 AD (AT/AA), 5 AD (AT), 6 AD (TT/AA), 7 AD (TT/GG), 8 C (TT/GG), 9 C (AT/AG). Exposure time in lanes 1 and 2 was 5 sec and in lanes 3–9, 7 min. (C) Immunoprecipitates of E11 (4 μg) (lanes 3, 5, 7) or IgG1 (4 μg) (lanes 2, 4, 6) with sCR1 (2 μg) (lanes 2,3), RBC from a MCI subject (TT/GG) (50 μg protein, lane 4, 5) or brain extracts from cognitively intact subject (AT/AG) (250 μg, lane 6, 7). sCR1 is loaded at 1 ng (lane 1). (D) Representative immunoprecipitations of brain lysates C (TT/GG) (250 μg protein) (lanes 3–6) with 8C9.1 (lane 4), J3B11 (lane 6), or corresponding isotype IgG (lanes 3, 5). Control sCR1 loaded at 1 ng (lane 1) and RBC extract loaded at 1 ng CR1 (lane 2). C and D. Blots were probed with polyclonal anti-CR1 Ab.

The ability of the different Abs tested by IHC to recognize CR1 by WB of brain and erythrocyte extracts was assessed. Clone E11 recognized sCR1 and both the smaller isoform (“F” in Fig 1B) of CR1 and the larger one (S) expected for CR1 SNP rs6656401 [16,37] in extracts of RBC (Fig 7A) by WB. In a few cases discrepancies between the size isoform and that expected from the SNP genotype (Fig 7A, lane 5) were noted (14%), as has been reported by others [37]. [Discordant samples were reanalyzed by WB to confirm CR1 size.] In contrast, when brain lysates were probed, E11 detected a band with single CR1 size. While the band did not match the expected size for samples known to have a least one copy of the rs6656401 SNP (Fig 7B), the reactivity disappeared when probed with E11 mAb preabsorbed with sCR1 (data not shown). Under reducing conditions, the H-2 mAb also recognized one band consistent with CR1 size. In contrast, the antibodies that were positive in IHC, J3B11 and 8C9.1, did not recognize the denatured receptor by WB of brain lysates (data not shown, and S1 Table).

These anti-CR1 Abs were then tested for their ability to IP CR1 from brain. All 3 mAbs tested were able to IP recombinant soluble CR1 and CR1 from RBC extracts since bands of the correct size were observed after being probed with pAb (Fig 7C and data not shown). However, E11 (Fig 7C, lane 7), J3B11 and 8C9.1 (Fig 7D, lanes 4 and 6) did not IP detectable CR1 from brain (even when starting with 250 μg of brain protein extract).

### sCR1 level in plasma varies slightly by CR1 polymorphisms but not by cognitive diagnosis

**Fig 8:**
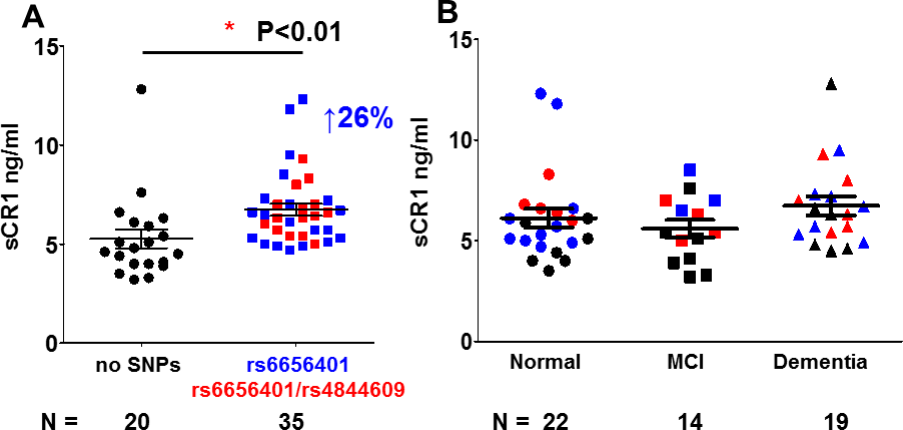
Soluble CR1 concentration (ng/ml) in plasma from donors with different CR1 SNPs and diagnosis. Soluble CR1 was measured by ELISA and plotted vs. A. SNP [rs6656401 only (n = 21) or rs6656401 and rs4844609 (n = 16) and without the rs6656401 and rs4844609 SNPs (n = 22)] or B. categorized by diagnosis [nondemented (n = 22),), MCI (n = 14) and AD (n = 19)]. Recombinant CR1 was used to generate the standard curve. Black symbols indicate samples with no variant SNP (rs6656401 and rs4844609), red reflects donors with two SNPs, and blue is donors with rs6656401 only. Data points are from multiple experiments in which samples were run in triplicate and normalized to standard CR1 samples across experiments.

Soluble CR1 concentration in plasma from 55 donors was detected with a range of 3.2 to 12.8 ng/ml. There was a slight but significant increase (24%) in sCR1 concentration that correlated with rs4844609 and rs6656401 risk alleles (p<0.03) versus those donors with the non risk CR1 alleles (Fig 8A). Plasma sCR1 concentration did not correlate with diagnosis (Fig 8B).

### CR1 containing the rs4844609 SNP differs in relative binding activity for C1q, while the slow form of CR1 is associated with higher C3b binding than the fast form

The rs4844609 SNP, occurring in previous study populations with a frequency of ~ 0.02, is associated with memory decline in at least some cohorts [16,38]. It is the only SNP thus far found to be associated with AD that is in a CR1 coding region (results in the substitution Ser1610Thr) and is located in CR1 LHR-D [16], the region reported to contain C1q/MBL binding sites [19] (Fig 1). Given that CR1 may mediate the clearance of C1q and/or C3b bound Aβ42 fibrils [25], this alteration may influence the efficiency of opsonized amyloid clearance. Therefore, we investigated if CR1 partially purified from RBC of donors containing the rs4844609 (and rs6656401) SNP showed altered C1q-CR1 binding and/or C3b-CR1 binding.

**Fig 9:**
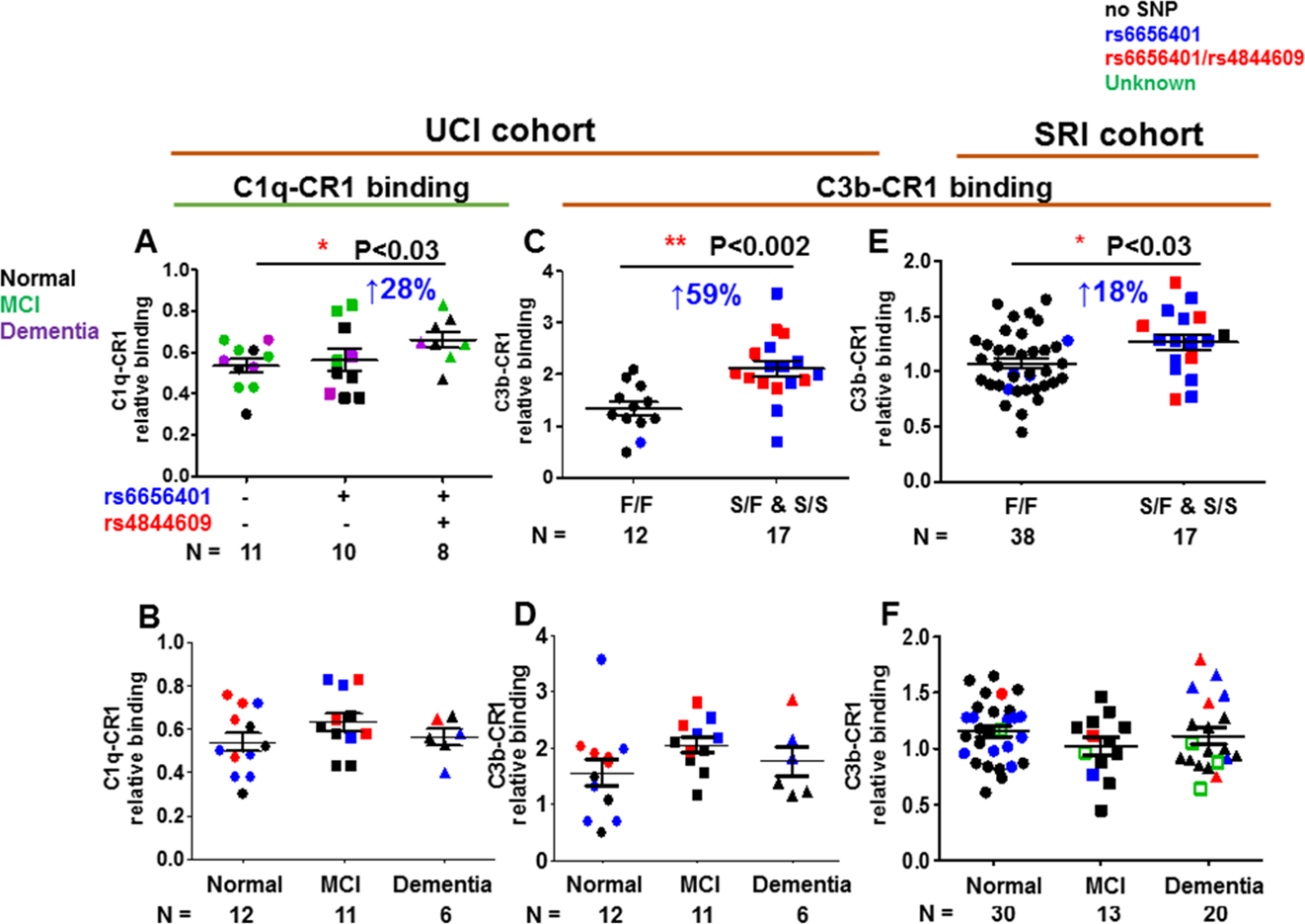
C1q-CR1 and C3b-CR1 relative binding activity. CR1 partially purified from RBC was assessed for binding to immobilized C1q (A, B) or C3b (C, D, E, F). A. UCI cohort: CR1- C1q relative binding for CR1with rs6656401 (n = 10) only, with rs6656401 and rs4844609 (n = 8), or without rs6656401 or rs4844609 (n = 11) SNPs. Black, green and purple symbols indicate patients clinically diagnosed as cognitively normal, mild cognitive impairment (MCI), and demented (AD) respectively. B. UCI cohort: CR1-C1q relative binding vs diagnosis: Normal (n = 12), MCI (n = 11) and Dementia (n = 16). In C and E, CR1-C3b relative binding is plotted by CR1 isoform (determined by WB): donors with common allele (fast form or F form) (n = 12) or with extra C3b-binding domain (n = 17) (slow form or S form) in UCI cohort (C) or F form (n = 38), S form (n = 17) for SRI cohort (E). In D and F, CR1-C3b relative binding plotted vs diagnosis: Normal (n = 12), MCI (n = 11), Dementia (n = 6) in UCI cohort (D) or normal (n = 30), MCI (n = 13), Dementia (n = 20) in SRI cohort (F). (Reference CR1 samples were used to standardize values among assays performed on different dates). In B-F: samples with no variant SNP (black); red, donors with two SNPs (rs6656401 or rs4844609); blue, donors with rs6656401 only and green open square is donor with unknown SNP genotype. Subjects in whom the CR1 SNP analysis was discordant with the F/F WB results are visible as blue circles in Fig 9C and E. *p<0.01–0.05, **p<0.01–0.001, calculated using 1 way ANOVA. Data points are from multiple experiments in which samples were run in triplicate and normalized to standard CR1 samples across experiments.

CR1 from individuals with at least one copy of both rs4844609 and rs6656401 SNPs (n = 8) showed slightly higher C1q-CR1 relative binding activity (28%, p<0.03) than the CR1 without either SNP (n = 11) (Fig 9A). However, the CR1 with only rs6656401 SNP (n = 10) did not affect C1q/CR1 binding indicating that only rs4844609 SNP (located in the C1q binding domain) is critical for the increased binding effect seen (Fig 9A and data not shown).

Interestingly, CR1 with the slow (S) form and the rs6656401 SNP, with or without the rs4844609 SNP (n = 17), showed a significantly higher C3b-CR1 relative binding activity (59%, p<0.002) than CR1 with only the fast (F) form (common allele) (n = 12) (Fig 9C) in the UCI cohort. Similarly, in a second cohort (SRI), C3b-CR1 binding activity was slightly but significantly higher (18%, p<0.03) with the S form of CR1 (n = 17) than with the F form of CR1 only (n = 38). [It can be noted that there were four subjects in whom the CR1 SNP analysis was discordant with WB results in the UCI cohort. However, this discordance resulted in only one subject in the F/F CR1 category (Fig 9C, blue circle). There was also 14% discordancy in the SRI cohort (9 out of 64). Four subjects with the rs6656401 CR1 SNP that normally associates with the S form of CR1, showed only the F CR1 isoform category (Fig 9E, blue circles). All of these samples displayed binding characteristics of the isoform detected by WB.] Diagnosis was not associated with higher or lower C1q/CR1 relative binding (Fig 9B) or C3b/CR1 relative binding (Fig 9D and 9F).

## Discussion

Detrimental and neuroprotective effects of the complement system have been shown in Alzheimer’s disease as well as other neurodegenerative diseases (reviewed in [39,40]. Fibrillar (ß-sheet) amyloid plaques can activate complement generating C5a that recruit activated glia to the plaque. These activated cells can secrete proinflammatory cytokines or other toxic mediators that can enhance neurodegeneration. The detrimental role of C1 has been demonstrated A transgenic model of AD deficient in C1q, and therefore unable to activate complement via the classical pathway, showed less pathology relative to the C1q-sufficient wild type [41]. In addition, treatment of a transgenic model of AD with an antagonist of C5aR1 improved pathology and behavior [42], suggesting that the detrimental consequences of complement activation were due to the proinflammatory C5a activation fragment. In prion disease, another neurodegenerative disorder, complement factors such as C1q and C3b were shown to be associated with prion deposits and the membrane attack complex was detected on neurons suggesting detrimental complement activation in brain [43]. Furthermore, recent studies in a mouse model of chronic wasting disease showed that CD21/CD35 mediates splenic prion trapping, accumulation and replication that leads to neuropathology [44,45]

In contrast, C3 has been shown to have beneficial effects in a mouse model of AD [46], which may be due to the enhancement of clearance of C3b-opsonized amyloid [47]. In addition, C1q induces neuroprotective activities, by inducing neuron survival and neuroprotective gene pathways [48–50], binding to apoptotic neurons and cellular debris inducing their clearance and modulating microglial proinflammatory cytokine expression ([51,52] and reviewed in [39]. These may be particularly important at early stages of injury prior to induction of synthesis of other complement components. Finally, C1 and C3b mediate synaptic pruning [53] which during aging appears to be detrimental, but during development aids in proper synaptic segregation. Thus, the role of complement in brain is clearly complex and defined by age, genetics and injury.

Recent studies have found associations of the CR1 SNPs rs6656401 and rs4844609 with AD and cognitive decline [3,15,16], although there is some controversy with respect to the association of memory decline with coding variant rs4844609, Ser1610Thr [30]. CR1 SNP rs3818361 was also reported to associate with AD but only in subjects also carrying the ApoE4 genotype [3], withApoE4 carriers having lower brain amyloid burden than non-ApoE4 carriers at the time of autopsy. However, the functional consequence of any of these associations remains unknown. In the present work, we have assessed anti-CR1 reactivity in brain as well as C1q and C3b binding to CR1 isolated from RBC of control or AD patients, with or without the GWAS-reported CR1 SNPs rs6656401and rs4844609, in order to determine if the diagnosis or SNPs would have an effect on CR1 distribution or function. CR1 immunoreactivity that was blocked by preabsorption of reactive Abs with human soluble recombinant CR1 was localized to astrocytes (in addition to red blood cells remaining in the brain vasculature), but the diagnosis or the presence of the SNPs did not affect the distribution or abundance of the receptor detected. The presence of anti-CR1 reactivity in astrocytes was confirmed by colocalization with GFAP and by immunocytochemistry using human adult brain-derived astrocyte cultures. Gasque and colleagues reported CR1 localization in astrocytes in human brains from normal or multiple sclerosis patients as well as in human primary astrocyte cell cultures or cell lines [54]. However, others have reported CR1 mRNA expression in phagocytic Kolmer cells of the choroid plexus (by in situ hybridization) [55], anti-CR1 reactivity on brain Kolmer and ependymal cells though only during bacterial meningitis [56], and anti-CR1 staining of microglia and neurons [57,58], none of which we were able to replicate in our normal, MCI or AD brains or in human brain derived microglial cultures. The differences in results seen among the studies could be due to differences in Ab reactivity or levels of detection. In addition, Ab specificity by preabsorption of the anti-CR1 with sCR1 was not demonstrated by others. In our hands, neuronal staining was not blocked by preabsorption with sCR1, whereas astrocyte staining was, consistent with specific staining on astrocytes but nonspecific labelling of neurons. One other possibility is that both J3B11 and 8C9.1 (though they recognize different epitopes in CR1) react with some other molecule in brain that happens to be blocked by preabsorption with soluble recombinant CR1. However, these antibodies do not react with human CR2 by ICC or WB analysis.

CR1 structural polymorphisms are well characterized [11]. There are four isoforms with different lengths (Fig 1). The F (fast migration) and S (slow migration) forms are more common in the population (83 and 15% respectively) [11]. The longer alleles (S) were reported to be risk alleles for AD [15]. The SNP rs6656401 is usually found associated with the S form that has an additional LHR and thus an additional C3b/C4b binding site [16]. Our WB analyses with RBC from patients with different genotypes have confirmed a general association of rs6656401 with the S form, although there was some discordance (14%) between genotypes and structural isoforms as also observed in another recent study [37]. However, when brain lysates were assessed by WB analysis only 2 Abs (E11 and H-2) positively stained a band with a size consistent with CR1. This one band was detected in all samples regardless of genotype. Immunoprecipitation of CR1 from brain lysates was also negative. These results suggest that either an alternative or modified form of CR1 is expressed in brain, or that CR1 is expressed at a level below the detection of our antibodies by WB. One previous report indicated that the S form is present at lower levels than the F form in brain [57].

SNP rs4844609 [16] is located in the LHR-D region of CR1, the LHR closest to the membrane and the cleavage site that generates sCR1 [27,29], a soluble molecule known to have complement inhibitory activity. Enhanced susceptibility of that site to cleavage could lead to less regulation of complement on the surface of host cells while a decrease in the release of the soluble form could lead to lower regulation of the cascade in the fluid state. Elevated levels of plasma sCR1 have been reported in some diseases [59], but lower levels were found in other conditions [60]. In a small cohort of patients (171) Daborg and colleagues reported a slight elevation in CR1 in cerebrospinal fluid of individuals with AD or MCI that developed AD relative to controls plus those with MCI that had not advanced to AD [61]. We observed a small but statistically significant increase in sCR1 concentration in plasma that correlated with the presence of rs4844609 or/and rs6656401 compared to donors with the common CR1 alleles consistent with a slight alteration in the efficiency of cleavage of CR1 by the presence of the SNPs. However, given the small size of the effect, thus far, there is no evidence to support a link between plasma sCR1 levels and dementia in this small cohort.

The colocalization of C3b and CR1 with Aß at the RBC surface is consistent with a role for CR1-mediated clearance of Aß from blood [25] similar to the role of immune adherence in clearing immune complexes from the blood. Therefore, it is possible that the presence of either SNP could alter the clearance of C1q or C3b opsonized amyloid. The LHR-D region containing the rs4844609 SNP (resulting in a change of Ser 1610 to Thr) also contains C1q and ficolin/MBL binding sites [10,19]. CR1 isolated from donors homozygous or heterozygous for rs4844609 showed a small but significant increase in C1q binding compared to those without the risk rs4844609 allele. It remains to be seen if this increase (although statistically significant) is physiologically significant. C3b binding to CR1 was significantly increased in the genotypes containing at least one slow (S) form allele with the rs6656401 SNP compared to genotypes with only the F isoform allele. The increase in C3b binding observed is consistent with the presence of an extra C3b binding site in the S form (4 vs. 3) although differences in relative binding may be underestimated given the dominant contribution of F isoforms over the S isoform in the purified CR1 samples due to inherently greater copy number levels of the F form on RBC. Clearly more precise measurement of C1q and C3b binding to purified isoforms of CR1 will be required to determine the quantitative differences in both binding and processing of Aß-C3b complexes that result from the SNP in CR1. How this slightly higher binding activity would lead to a detrimental effect as is suggested by the SNP association with AD remains to be seen.

Alternatively, the fact that in most, but not all, families there are much lower levels of the S form of CR1 expressed on RBC relative to the F form [62] (and reviewed in [13]), may account for a lower capacity for peripheral clearance of Aß by RBC expressing the S form of CR1 (and thus a risk form), regardless of the relative binding activity, which would exacerbate the reduced expression of CR1 on E that occurs with aging [63]. If clearance of peripheral Aß diminishes Aß in the brain, the so called “peripheral sink model” [64,65], a deficiency in such peripheral clearance due to lower density of CR1 on E, could contribute to the association of the S form linked SNPs with cognitive impairment. Since the role of peripheral clearance in reducing brain Aß has been recently challenged [66], a better understanding of the relationship of this process to cognitive impairment is needed.

In conclusion, our results show that CR1 distribution in brain is not correlated with the SNPs rs4844609 or rs6656401 or diagnosis. C1q and C3b binding to CR1 are only moderately modified by the SNP rs4844609 or the S form (which predominantly is associated with rs6656401) respectively. Nevertheless, the lower level of erythrocyte expression seen with these GWAS-identified variants associated with AD [37] may reduce the efficiency of peripheral clearance of amyloid ß, and thus lead to less efficient clearance from the brain. Further functional studies are needed to determine if, and to what extent, RBC CR1 mediated clearance of peripheral Aß contributes to the association of these polymorphisms with susceptibility to AD, or if another functional mechanism is involved. Identification of the molecular pathways involved will reveal targets for therapeutic intervention.

## Supporting Information

S1 Fig CR1 is not detected on microglia in human brain tissue. Immunofluorescent dual labelling of 8C9.1 (green) and Iba1 (red) in frontal cortex of AD case. Scale bar: 50 um.
(TIF)

S1 Table Summary of CR1 antibody reactivity. (PDF)

## Abbreviations

Ab: antibody
AD: Alzheimer’s disease
BSA: bovine serum albumin
CR1: Complement receptor one (aka, CD35, C3b/C4b receptor, immune adherence receptor
fAß: fibrillar amyloid beta peptide
GFAP: glial fibrillary acidic protein
GWAS: genome wide association studies
mAb: monoclonal antibody
MCI: mild cognitive impairment
SNP: single nucleotide polymorphism

## References

[1] Ricklin D, Lambris JD (2013) Complement in immune and inflammatory disorders: pathophysiological mechanisms. J Immunol 190: 3831–3838. 190/8/3831 [pii]; 10.4049/jimmunol.1203487 23564577 PMC3623009

[2] Sjoberg AP, Trouw LA, Blom AM (2009) Complement activation and inhibition: a delicate balance. Trends Immunol 30: 83–90. S1471-4906(08)00271-8 [pii]; 10.1016/j.it.2008.11.003 19144569

[3] Lambert JC, Heath S, Even G, Campion D, Sleegers K, Hiltunen M, et al (2009) Genome-wide association study identifies variants at CLU and CR1 associated with Alzheimer's disease. Nat Genet 41: 1094–1099. [pii]; 10.1038/ng.439 19734903

[4] Carrasquillo MM, Belbin O, Hunter TA, Ma L, Bisceglio GD, Zou F, et al (2010) Replication of CLU, CR1, and PICALM associations with alzheimer disease. Arch Neurol 67: 961–964. [pii]; 10.1001/archneurol.2010.147 20554627 PMC2919638

[5] Jun G, Naj AC, Beecham GW, Wang LS, Buros J, Gallins PJ, et al (2010) Meta-analysis confirms CR1, CLU, and PICALM as alzheimer disease risk loci and reveals interactions with APOE genotypes. Arch Neurol 67: 1473–1484. [pii]; 10.1001/archneurol.2010.201 20697030 PMC3048805

[6] Corneveaux JJ, Myers AJ, Allen AN, Pruzin JJ, Ramirez M, Engel A, et al (2010) Association of CR1, CLU and PICALM with Alzheimer's disease in a cohort of clinically characterized and neuropathologically verified individuals. Hum Mol Genet 19: 3295–3301. [pii]; 10.1093/hmg/ddq221 20534741 PMC2908469

[7] Zhang B, Gaiteri C, Bodea LG, Wang Z, McElwee J, Podtelezhnikov AA, et al (2013) Integrated Systems Approach Identifies Genetic Nodes and Networks in Late-Onset Alzheimer's Disease. Cell 153: 707–720. S0092-8674(13)00387-5 [pii]; 10.1016/j.cell.2013.03.030 23622250 PMC3677161

[8] Villiers MB, Villiers CL, Jacquier-Sarlin MR, Gabert FM, Journet AM, Colomb MG (1996) Covalent binding of C3b to tetanus toxin: influence on uptake/internalization of antigen by antigen-specific and non-specific B cells. Immunology 89: 348–355. 8958046 10.1046/j.1365-2567.1996.d01-747.x PMC1456555

[9] Khera R, Das N (2009) Complement Receptor 1: disease associations and therapeutic implications. Mol Immunol 46: 761–772. 10.1016/j.molimm.2008.09.026 19004497 PMC7125513

[10] Jacquet M, Lacroix M, Ancelet S, Gout E, Gaboriaud C, Thielens NM, et al (2013) Deciphering complement receptor type 1 interactions with recognition proteins of the lectin complement pathway. J Immunol 190: 3721–3731. [pii]; 10.4049/jimmunol.1202451 23460739

[11] Krych-Goldberg M, Atkinson JP (2001) Structure-function relationships of complement receptor type 1. Immunological Reviews 180: 112–122. 11414353 10.1034/j.1600-065x.2001.1800110.x

[12] Aiyaz M, Lupton MK, Proitsi P, Powell JF, Lovestone S (2012) Complement activation as a biomarker for Alzheimer's disease. Immunobiology 217: 204–215. S0171-2985(11)00163-X [pii]; 10.1016/j.imbio.2011.07.023 21856034

[13] Liu D, Niu ZX (2009) The structure, genetic polymorphisms, expression and biological functions of complement receptor type 1 (CR1/CD35). Immunopharmacol Immunotoxicol 31: 524–535. 10.3109/08923970902845768 19874218

[14] Tetteh-Quarcoo PB, Schmidt CQ, Tham WH, Hauhart R, Mertens HD, Rowe A, et al (2012) Lack of evidence from studies of soluble protein fragments that Knops blood group polymorphisms in complement receptor-type 1 are driven by malaria. PLoS ONE 7: e34820 10.1371/journal.pone.0034820;PONE-D-12-01197 [pii]. 22506052 PMC3323580

[15] Brouwers N, Van CC, Engelborghs S, Lambert JC, Bettens K, Le BN, et al (2012) Alzheimer risk associated with a copy number variation in the complement receptor 1 increasing C3b/C4b binding sites. Mol Psychiatry 17: 223–233. mp201124 [pii]; 10.1038/mp.2011.24 21403675 PMC3265835

[16] Keenan BT, Shulman JM, Chibnik LB, Raj T, Tran D, Sabuncu MR, et al (2012) A coding variant in CR1 interacts with APOE-epsilon4 to influence cognitive decline. Hum Mol Genet 21: 2377–2388. [pii]; 10.1093/hmg/dds054 22343410 PMC3335317

[17] Nickells M, Hauhart R, krych M, Subramanian VB, Geoghegan-Barek K, Marsh HC Jr, et al (1998) Mapping epitopes for 20 monoclonal antibodies to CR1. Clin exp Immunol 112: 27–33. 9566786 10.1046/j.1365-2249.1998.00549.x PMC1904933

[18] Moulds JM, Brai M, Cohen J, Cortelazzo A, Cuccia M, Lin M, et al (1998) Reference typing report for complement receptor 1 (CR1). Exp Clin Immunogenet 15: 291–294. 19084 [pii];19084. 10072640 10.1159/000019084

[19] Klickstein LB, Barbashov SF, Liu T, Jack RM, Nicholson-Weller A (1997) Complement receptor type 1 (CR1, CD35) is a receptor for C1q. Immunity 7: 345–355. 9324355 10.1016/s1074-7613(00)80356-8

[20] Larvie M, Shoup T, Chang WC, Chigweshe L, Hartshorn K, White MR, et al (2012) Mannose-binding lectin binds to amyloid beta protein and modulates inflammation. J Biomed Biotechnol 2012: 929803 10.1155/2012/929803 22536027 PMC3322523

[21] Afagh A, Cummings BJ, Cribbs DH, Cotman CW, Tenner AJ (1996) Localization and cell association of C1q in Alzheimer's disease brain. Exp Neurol 138: 22–32. S0014-4886(96)90043-6 [pii]; 10.1006/exnr.1996.0043 8593893

[22] Rogers J, Cooper NR, Webster S, Schultz J, McGeer PL, Styren SD, et al (1992) Complement activation by beta-amyloid in Alzheimer disease. Proc Natl Acad Sci 89: 10016–10020. 1438191 10.1073/pnas.89.21.10016 PMC50268

[23] Velazquez P, Cribbs DH, Poulos TL, Tenner AJ (1997) Aspartate residue 7 in amyloid beta-protein is critical for classical complement pathway activation: implications for Alzheimer's disease pathogenesis. Nat Med 3: 77–79. 8986745 10.1038/nm0197-77

[24] Bradt BM, Kolb WP, Cooper NR (1998) Complement-dependent proinflammatory properties of the Alzheimer's disease beta-peptide. J Exp Med 188: 431–438. 9687521 10.1084/jem.188.3.431 PMC2212467

[25] Rogers J, Li R, Mastroeni D, Grover A, Leonard B, Ahern G, et al (2006) Peripheral clearance of amyloid beta peptide by complement C3-dependent adherence to erythrocytes. Neurobiol Aging 27: 1733–1739. S0197-4580(05)00313-1 [pii]; 10.1016/j.neurobiolaging.2005.09.043 16290270

[26] Tas SW, Klickstein LB, Barbashov SF, Nicholson-Weller A (1999) C1q and C4b bind simultaneously to CR1 and additively support erythrocyte adhesion. J Immunol 163: 5056–5063. 10528211

[27] Chen CH, Ghiran I, Beurskens FJ, Weaver G, Vincent JA, Nicholson-Weller A, et al (2007) Antibody CR1-2B11 recognizes a non-polymorphic epitope of human CR1 (CD35). Clin exp Immunol 148: 546–554. CEI3355 [pii]; 10.1111/j.1365-2249.2007.03355.x 17493021 PMC1941935

[28] Weisman HF, Bartow T, Leppo MK, Marsh HC Jr, Carson GR, Concino MF, et al (1990) Soluble human complement receptor type 1: In vivo inhibitor of complement suppressing post-ischemic myocardial inflammation and necrosis. Science (New York, N Y) 249: 146–151. 10.1126/science.2371562 2371562

[29] Sadallah S, Hess C, Miot S, Spertini O, Lutz H, Schifferli JA (1999) Elastase and metalloproteinase activities regulate soluble complement receptor 1 release. Eur J Immunol 29: 3754–3761. 10556832 10.1002/(SICI)1521-4141(199911)29:11<3754::AID-IMMU3754>3.0.CO;2-5

[30] Van Cauwenberghe C, Bettens K, Engelborghs S, Vandenbulcke M, Van DJ, Vermeulen S, et al (2013) Complement receptor 1 coding variant p.Ser1610Thr in Alzheimer's disease and related endophenotypes. Neurobiol Aging 34: 2235–2236. S0197-4580(13)00108-5 [pii]; 10.1016/j.neurobiolaging.2013.03.008 23582656

[31] Lue LF, Brachova L, Walker DG, Rogers J (1996) Characterization of glial cultures from rapid autopsies of Alzheimer's and control patients. Neurobiol Aging 17: 421–429. 0197458096000061 [pii]. 8725904 10.1016/0197-4580(96)00006-1

[32] Makrides SC, Scesney SM, Ford PJ, Evans KS, Carson GR, Marsh HC (1992) Cell surface expression of the C3b/C4b receptor (CR1) protects Chinese hamster ovary cells from lysis by human complement. J Biol Chem 267: 24754–24761. 1447213

[33] Raff MC, Abney ER, Cohen J, Lindsay R, Noble M (1983) Two types of astrocytes in cultures of developing rat white matter: differences in morphology, surface gangliosides, and growth characteristics. J Neurosci 3: 1289–1300. 6343560 10.1523/JNEUROSCI.03-06-01289.1983 PMC6564607

[34] Leonard BW, Mastroeni D, Grover A, Liu Q, Yang K, Gao M, et al (2009) Subventricular zone neural progenitors from rapid brain autopsies of elderly subjects with and without neurodegenerative disease. J Comp Neurol 515: 269–294. 10.1002/cne.22040 19425077 PMC2757160

[35] van den Berge SA, Middeldorp J, Zhang CE, Curtis MA, Leonard BW, Mastroeni D, et al (2010) Longterm quiescent cells in the aged human subventricular neurogenic system specifically express GFAP-delta. Aging Cell 9: 313–326. ACE556 [pii]; 10.1111/j.1474-9726.2010.00556.x 20121722

[36] Berger M, O'Shea J, Cross AS, Folks TM, Chused TM, Brown EJ, et al (1984) Human neutrophils increase expression of C3bi as well as C3b receptors upon activation. J Clin Invest 74: 1566–1571. 10.1172/JCI111572 6209300 PMC425333

[37] Mahmoudi R, Kisserli A, Novella JL, Donvito B, Drame M, Reveil B, et al (2015) Alzheimer's disease is associated with low density of the long CR1 isoform. Neurobiol Aging 36: 1766–12. S0197-4580(15)00037-8 [pii]; 10.1016/j.neurobiolaging.2015.01.006 25666996

[38] Chibnik LB, Shulman JM, Leurgans SE, Schneider JA, Wilson RS, Tran D, et al (2011) CR1 is associated with amyloid plaque burden and age-related cognitive decline. Ann Neurol 69: 560–569. 10.1002/ana.22277 21391232 PMC3066288

[39] Alexander JJ, Anderson AJ, Barnum SR, Stevens B, Tenner AJ (2008) The complement cascade: Yin-Yang in neuroinflammation—neuro-protection and -degeneration. J Neurochem 107: 1169–1187. 10.1111/j.1471-4159.2008.05668.x 18786171 PMC4038542

[40] Wyss-Coray T, Rogers J (2012) Inflammation in Alzheimer disease-a brief review of the basic science and clinical literature. Cold Spring Harb Perspect Med 2: a006346 10.1101/cshperspect.a006346 22315714 PMC3253025

[41] Fonseca MI, Zhou J, Botto M, Tenner AJ (2004) Absence of C1q leads to less neuropathology in transgenic mouse models of Alzheimer's disease. J Neurosci 24: 6457–6465. 15269255 10.1523/JNEUROSCI.0901-04.2004 PMC6729885

[42] Fonseca MI, Ager RR, Chu SH, Yazan O, Sanderson SD, LaFerla FM, et al (2009) Treatment with a C5aR antagonist decreases pathology and enhances behavioral performance in murine models of Alzheimer's disease. J Immunol 183: 1375–1383. [pii]; 10.4049/jimmunol.0901005 19561098 PMC4067320

[43] Kovacs GG, Gasque P, Strobel T, Lindeck-Pozza E, Strohschneider M, Ironside JW, et al (2004) Complement activation in human prion disease. Neurobiol Dis 15: 21–28. 14751767 10.1016/j.nbd.2003.09.010

[44] Michel B, Ferguson A, Johnson T, Bender H, Meyerett-Reid C, Pulford B, et al (2012) Genetic depletion of complement receptors CD21/35 prevents terminal prion disease in a mouse model of chronic wasting disease. J Immunol 189: 4520–4527. [pii]; 10.4049/jimmunol.1201579 23002439 PMC3478448

[45] Mabbott NA, Bruce ME, Botto M, Walport MJ, Pepys MB (2001) Temporary depletion of complement component C3 or genetic deficiency of C1q significantly delays onset of scrapie. Nat Med 7: 485–487. 11283677 10.1038/86562

[46] Maier M, Peng Y, Jiang L, Seabrook TJ, Carroll MC, Lemere CA (2008) Complement C3 deficiency leads to accelerated amyloid beta plaque deposition and neurodegeneration and modulation of the microglia/macrophage phenotype in amyloid precursor protein transgenic mice. J Neurosci 28: 6333–6341. 10.1523/JNEUROSCI.0829-08.2008 18562603 PMC3329761

[47] Shi Q, Colodner KJ, Matousek SB, Merry K, Hong S, Kenison JE, et al (2015) Complement C3-Deficient Mice Fail to Display Age-Related Hippocampal Decline. J Neurosci 35: 13029–13042. 35/38/13029 [pii]; 10.1523/JNEUROSCI.1698-15.2015 26400934 PMC6605437

[48] Benoit ME, Hernandez MX, Dinh ML, Benavente F, Vasquez O, Tenner AJ (2013) C1q-induced LRP1B and GPR6 Proteins Expressed Early in Alzheimer Disease Mouse Models, Are Essential for the C1q-mediated Protection against Amyloid-beta Neurotoxicity. J Biol Chem 288: 654–665. [pii]; 10.1074/jbc.M112.400168 23150673 PMC3537064

[49] Benoit ME, Tenner AJ (2011) Complement protein C1q-mediated neuroprotection is correlated with regulation of neuronal gene and microRNA expression. J Neurosci 31: 3459–3469. 31/9/3459 [pii]; 10.1523/JNEUROSCI.3932-10.2011 21368058 PMC3080046

[50] Pisalyaput K, Tenner AJ (2008) Complement component C1q inhibits beta-amyloid- and serum amyloid P-induced neurotoxicity via caspase- and calpain-independent mechanisms. J Neurochem 104: 696–707. 17986223 10.1111/j.1471-4159.2007.05012.x

[51] Fraser DA, Pisalyaput K, Tenner AJ (2010) C1q enhances microglial clearance of apoptotic neurons and neuronal blebs, and modulates subsequent inflammatory cytokine production. J Neurochem 112: 733–743. JNC6494 [pii]; 10.1111/j.1471-4159.2009.06494.x 19919576 PMC2809134

[52] Fraser DA, Laust AK, Nelson EL, Tenner AJ (2009) C1q differentially modulates phagocytosis and cytokine responses during ingestion of apoptotic cells by human monocytes, macrophages, and dendritic cells. J Immunol 183: 6175–6185. [pii]; 10.4049/jimmunol.0902232 19864605 PMC2843563

[53] Stevens B, Allen NJ, Vazquez LE, Howell GR, Christopherson KS, Nouri N, et al (2007) The classical complement cascade mediates CNS synapse elimination. Cell 131: 1164–1178. 18083105 10.1016/j.cell.2007.10.036

[54] Gasque P, Chan P, Mauger C, Schouft MT, Singhrao S, Dierich MP, et al (1996) Identification and characterization of complement C3 receptors on human astrocytes. J Immunol 156: 2247–2255. 8690915

[55] Singhrao SK, Neal JW, Rushmere NK, Morgan BP, Gasque P (1999) Differential expression of individual complement regulators in the brain and choroid plexus. Lab Invest 79: 1247–1259. 10532588

[56] Canova C, Neal JW, Gasque P (2006) Expression of innate immune complement regulators on brain epithelial cells during human bacterial meningitis. J Neuroinflammation 3: 22 [pii]; 10.1186/1742-2094-3-22 16948860 PMC1574292

[57] Hazrati LN, Van CC, Brooks PL, Brouwers N, Ghani M, Sato C, et al (2012) Genetic association of CR1 with Alzheimer's disease: a tentative disease mechanism. Neurobiol Aging 33: 2949 S0197-4580(12)00376-4 [pii]; 10.1016/j.neurobiolaging.2012.07.001 22819390

[58] Zanjani H, Finch CE, Kemper C, Atkinson J, McKeel D, Morris JC, et al (2005) Complement activation in very early Alzheimer disease. Alzheimer Dis Assoc Disord 19: 55–66. 15942322 10.1097/01.wad.0000165506.60370.94

[59] Pascual M, Duchosal MA, Steiger G, Giostra E, Pech+¿re A, Paccaud JP, et al (1993) Circulating soluble CR1 (CD35). Serum levels in diseases and evidence for its release by human leukocytes. J Immunol 151: 1702–1711. 8335953

[60] Karthikeyan G, Baalasubramanian S, Seth S, Das N (2007) Low levels of plasma soluble complement receptor type 1 in patients receiving thrombolytic therapy for acute myocardial infarction. J Thromb Thrombolysis 23: 115–120. 10.1007/s11239-006-9040-5 17131173

[61] Daborg J, Andreasson U, Pekna M, Lautner R, Hanse E, Minthon L, et al (2012) Cerebrospinal fluid levels of complement proteins C3, C4 and CR1 in Alzheimer's disease. J Neural Transm 119: 789–797. 10.1007/s00702-012-0797-8 22488444

[62] Dykman TR, Cole JL, Iida K, Atkinson JP (1983) Polymorphism of human erythrocyte C3b/C4b receptor. Proc Natl Acad Sci U S A 80: 1698–1702. 6572933 10.1073/pnas.80.6.1698 PMC393670

[63] Moldenhauer F, Botto M, Walport MJ (1988) The rate of loss of CR1 from ageing erythrocytes in vivo in normal subjects and SLE patients: no correlation with structural or numerical polymorphisms. Clin exp Immunol 72: 74–78. 2899464 PMC1541491

[64] Lemere CA, Spooner ET, LaFrancois J, Malester B, Mori C, Leverone JF, et al (2003) Evidence for peripheral clearance of cerebral Abeta protein following chronic, active Abeta immunization in PSAPP mice. Neurobiol Dis 14: 10–18. 13678662 10.1016/s0969-9961(03)00044-5

[65] Deane R, Bell RD, Sagare A, Zlokovic BV (2009) Clearance of amyloid-beta peptide across the blood-brain barrier: implication for therapies in Alzheimer's disease. CNS Neurol Disord Drug Targets 8: 16–30. 19275634 10.2174/187152709787601867 PMC2872930

[66] Walker JR, Pacoma R, Watson J, Ou W, Alves J, Mason DE, et al (2013) Enhanced proteolytic clearance of plasma Abeta by peripherally administered neprilysin does not result in reduced levels of brain Abeta in mice. J Neurosci 33: 2457–2464. 33/6/2457 [pii]; 10.1523/JNEUROSCI.3407-12.2013 23392674 PMC6619149

