# Supplementary figures and images for: Analysis of the Putative Role of CR1 in Alzheimer’s Disease: Genetic Association, Expression and Function
Source: PLoS One. 2016 Feb 25;11(2):e0149792. doi: 10.1371/journal.pone.0149792 (PMC4767815; doi:10.1371/journal.pone.0149792)

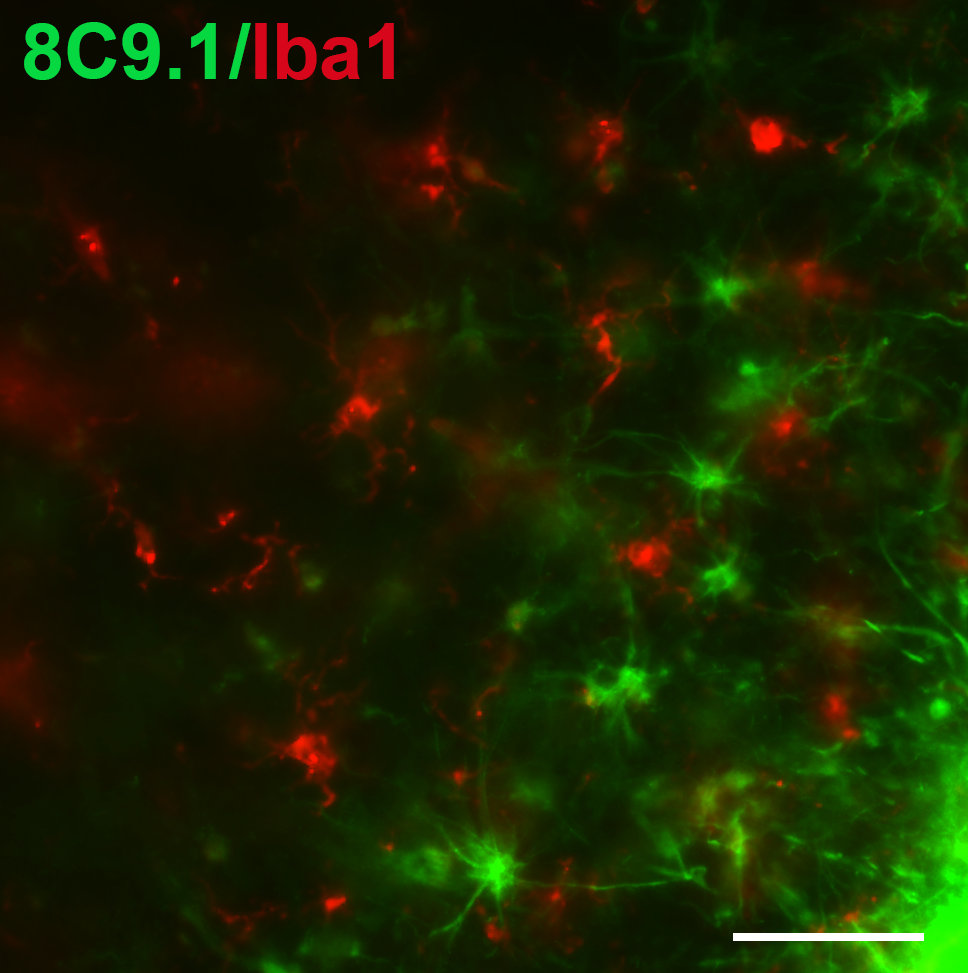

Supplement: S1 Fig — Immunofluorescent dual labelling of 8C9.1 (green) and Iba1 (red) in frontal cortex of AD case. Scale bar: 50 um. (TIF) [file pone.0149792.s001.tif]
