# Supplementary material for: Analysis of the Putative Role of CR1 in Alzheimer’s Disease: Genetic Association, Expression and Function
Source: PLoS One. 2016 Feb 25;11(2):e0149792. doi: 10.1371/journal.pone.0149792 (PMC4767815; doi:10.1371/journal.pone.0149792)
Supplement: S1 Table — (PDF) [file pone.0149792.s002.pdf]

Supplemental Table 1: Summary of CR1 antibody reactivity

Antibody	Source	IHC- Human Tissue			ICC		Western blot									IP		
		Cortex and hippocampus	sCR1 block	CP	CHO	Primary astros	sCR1		RBC		PMN		Brain		sCR1 block	sCR1	RBC	Brain
							R	NR	R	NR	R	NR	R	NR		R	R	R
**Monoclonal**																		
J3B11 (a)	Atkinson	Astros RBC	Yes	N	+	Yes		+ +		+				-		+	+	-
8C9.1 (b)	Atkinson	Astros neurons	Yes No	N	+	Yes		-		-						+	+	-
E11 (c)	Abcam	RBC Vessels?			+		+	+ +	+/-	+	-	+	+/-	+	Yes	+	+	-
H2 #166329 (d)	Santa Cruz	Neurons*	No		-			+		+			+	-				
#594708 (e)	R&D	N		N	+													
J3D3 (f)	Beckman	RBC Vessels?		N			+	+ +	-	+	-	+	-	-				
**Polyclonal**																		
pAb (g)	Atkinson	Neurons RBC Vessels?	No	N	+		+	+ +		+			-	-				
H-300 #20924 (h)	Santa Cruz	RBC Vessels?		N			+/-	+					-	-				

IHC:Immunohistochemistry, ICC: immunocytochemistry, IP: Immunoprecipitation, sCR1 block: absorption with sCR1 blocks staining, CP: choroid plexus, R:reduced, NR: non reduced, N: negative, vessels?:possibly on vessels (EC or pericytes?)

* hippocampus not investigated (In all other cases both cortex and hippocampus were tested).

a), b), c), e) : Nickells, et al, 1998; d), e), f), h) Hazrati et al,2012; g) Makrides et al,1992.
